# Association between dietary inflammatory index and Parkinson’s disease from National Health and Nutrition Examination Survey (2003–2018): a cross-sectional study

**DOI:** 10.3389/fnins.2023.1203979

**Published:** 2023-07-20

**Authors:** Zhaohao Zeng, Yanmei Cen, Lu Wang, Xiaoguang Luo

**Affiliations:** ^1^Department of Neurology, Shenzhen People’s Hospital, The Second Clinical Medical College, The First Affiliated Hospital, Jinan University, Southern University of Science and Technology, Shenzhen, Guangdong, China; ^2^The First Clinical Medical College of Jinan University, Guangzhou, Guangdong, China; ^3^The Guangdong Provincial Clinical Research Center for Geriatrics, Shenzhen Clinical Research Center for Geriatrics, Shenzhen People’s Hospital (The Second Clinical Medical College, Jinan University; The First Affiliated Hospital, Southern University of Science and Technology), Xiaogan, Hubei, China; ^4^Department of Neurology, The Central Hospital of Xiaogan, Xiaogan, Hubei, China

**Keywords:** Parkinson’s disease, dietary inflammatory index, nutrition, National Health and Nutrition Examination Survey, diet

## Abstract

**Objected:**

To explore the association between Parkinson’s disease (PD) and dietary inflammatory index (DII) scores in adults over 40 years old in the US.

**Method:**

Data were collected from the National Health and Nutrition Examination Survey (NHANES) conducted from 2003 to 2018. A total of 21,994 participants were included in the study. A weighted univariate and multivariable logistic regression analysis was performed to investigate the association between the DII and PD, in which continuous variables or categorical variables grouped by tertiles was used. The relationship between DII and PD has been further investigated using propensity score matching (PSM) and a subgroup analysis stratified based on DII and PD characteristics. Moreover, restricted cubic spline (RCS) analysis was conducted to examine whether there was a nonlinear association between DII and PD.

**Results:**

A total of 21,994 participants were obtained for statistical analysis, made up of 263 patients with PD and 21,731 participants without PD. Univariate and multivariable logistics regression analysis showed DII to be positively associated with PD before and after matching. Subgroup analysis revealed a statistical difference in non-Hispanic whites, but RCS analysis suggested that there was no nonlinear relationship between the DII and PD.

**Conclusion:**

For participants over 40 years of age, higher DII scores were positively correlated with PD. In addition, these results support the ability of diet to be used as an intervention strategy for managing PD.

## 1. Introduction

Known as the second most common chronic degenerative neurological disease after Alzheimer’s disease, PD affects millions of people around the world (Raj et al., 2021). With the increase in aging populations around the world, more and more elderly people will be affected by this disease, which has become a global public health problem. Increasing attention has been paid to the association between dietary intake and chronic diseases in recent years, and studies have shown that different diet patterns can impact physical health in different ways (Cena and Calder, 2020; Krznarić et al., 2021; Zhao et al., 2023). An inappropriate dietary pattern is closely related to increased inflammatory factors in PD, which are often caused by oxidative stress and chronic neuroinflammation (Liu et al., 2021a; Bisaglia, 2022). There has been some research suggesting that a Western diet increases inflammation factors, which are associated with a higher risk of PD, while Mediterranean diets are associated with lower ones, which have protective effects against PD onset and progression (Bonaccio et al., 2017; Naja et al., 2017; Bisaglia, 2022; Fox et al., 2022; Xu et al., 2022). The Dietary Inflammation Index is an effective method for measuring a diet’s inflammatory potential (Shivappa et al., 2014; Liu et al., 2021a). The higher the DII score, the greater the likelihood that the diet will promote inflammation, while the lower the DII score, the greater the likelihood that the diet will resist inflammation (Shivappa et al., 2014; Ruiz-Canela et al., 2016). A previous study have investigated the relationship between DII and prodromal Parkinson’s disease (pPD), and have found that an increase in DII score is associated with a higher probability and incidence of pPD (Balomenos et al., 2022). However, the study utilized risk marker scores of PD to calculate the probability of pPD and define its presence. It may therefore be helpful to further explore the relationship between DII and PD to develop future prevention or treatment methods for this disease. We investigated the association between dietary inflammation and PD by examining 80,241 participants from the NHANES from 2003 to 2018.

## 2. Methods

### 2.1. Study population and design

**Figure 1:**
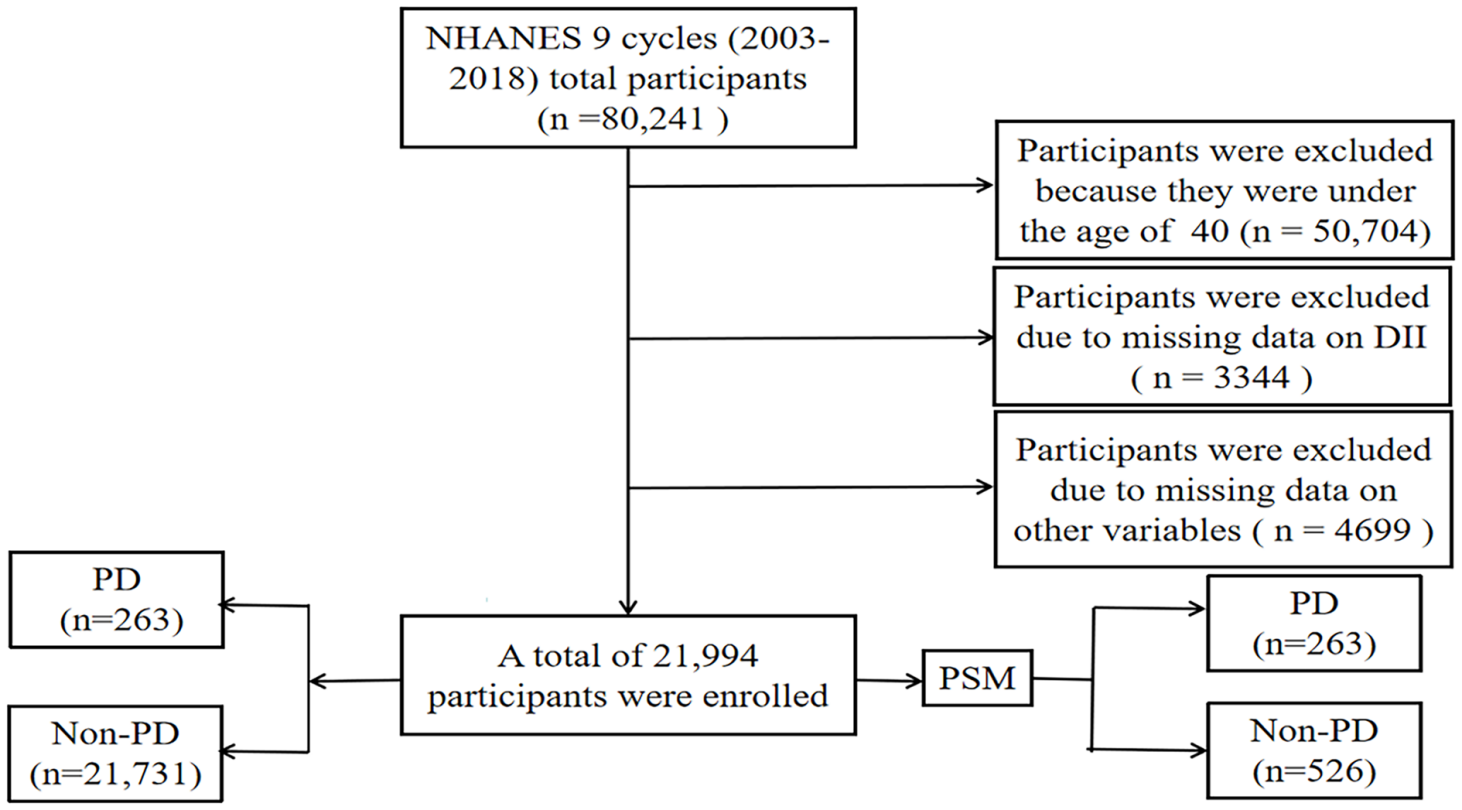
Flow chart of study inclusion legend: flowchart of the participants’ selection from NHANES 2003–2018.

In this study, nine cycles of NHANES data (2003–2018) were collected by the Centers for Disease Control and Prevention (CDC). NHANES is a multi-stage, stratified, large, nationally representative study of the United States population that provides detailed information about the study design, interviews, demographics, etc. (Woteki et al., 1988; Liu et al., 2021a; Gong et al., 2022). Among the sample of 80,241 adults, those who did not provide dietary information or other variables were excluded (Figure 1). Protocol approval was granted by the National Center for Health Statistics’ ethical review board, and informed consent forms were signed by participants.

### 2.2. Calculation of the dietary inflammation index

Based on a literature review, Shivappa developed the DII scoring system for evaluating the potential inflammation levels of dietary components (Shivappa et al., 2014; Liu et al., 2021a). Taking 45 nutrients into account, DII calculates the effects of dietary consumption on inflammation. To calculate DII, each component’s score is added together from the diet consumed over 24 h, including the proinflammatory and anti-inflammatory diet scores. In a literature review by Shivappa et al. (2014), the DII score calculation process is explained in detail. To calculate nutrient and energy-adjusted DII scores per 1,000 calories consumed, the energy density approach was used. It was the sum of each DII score that determined the participant’s DII score. There were 28/45 food parameters available for DII calculations in this study: carbohydrates, protein, total fat, alcohol, fiber, cholesterol, saturated fatty acids, monounsaturated fatty acids, polyunsaturated fatty acids, n-3 fatty acids, n-6 fatty acids, niacin, vitamin A, thiamine, vitamin B2, vitamin B6, vitamin B12, vitamin C, vitamin D, vitamin E, iron, magnesium, zinc, selenium, folic acid, carotene, caffeine, and energy. For pro-inflammatory diets, DII scores were higher, while for anti-inflammatory diets, DII scores were lower. The DII scores were still available even if the nutrients applied had been less than 30. To investigate the correlation between DII and PD, we categorized DII into tertiles based on baseline information of all participants, following the classification method used in previous studies (Liu et al., 2021a,b). Specifically, DII scores were divided into tertiles as follows: −5.281 to 0.818 for T1, 0.818 to 2.61 for T2, and 2.61 to 5.795 for T3.

### 2.3. Diagnosis of PD

In this study, participants were identified with PD by specifying “Second Level Category Name” as “ANTIPARKINSON AGENTS” in the Prescription Medications document. Based on the responses to questions regarding prescription medication, this determination was made. Since this method of identification was limited by medications and codes included in the NHANES, an individual had to be receiving treatment for PD to be classified as having it. Those who did not report taking an anti-parkinsonian medication were classified as not having PD. The definition of the disease is based on previous studies (DeMarco et al., 2021; Xu et al., 2022; Zhao et al., 2022).

### 2.4. Assessment of other variables

NHANES collects some sociodemographic information through structured data. Demographic covariates considered in the study include age, gender, race, education, and annual family income reported by interviewees. Measurements of waist size, weight, and height were conducted by well-trained health technologists following the anthropometry procedure manual. Body mass index (BMI) was calculated as weight (kg) divided by height squared (m2). Currently used clinical criteria and guidelines issued by the International Diabetes Association (IDM) were used to diagnose diabetes, with diabetes classified by either fasting blood glucose ≥7.0 mmol/L in laboratory tests, blood glucose >11.1 mmol/L in the OGTT experiment, those taking diabetes medications, those diagnosed with diabetes by their doctors during the survey, and those who self-reported (Dai et al., 2022). A stroke or heart attack was diagnosed based on the physician’s notification of whether the participant had a stroke or heart attack, as well as the physician’s notification (Zhao et al., 2023). Smoking status was categorized as “never” (<100 cigarettes in a lifetime), “former” (previous history of smoking but no longer smoking), or “now” (still smoking). Alcohol was categorized as “never” (never having drunk alcohol in a lifetime), “former” (previous history of drinking but no longer drinking), “heavy” alcohol use (≥3 drinks per day for females, ≥4 drinks per day for males, or binge drinking [≥4 drinks on the same occasion for females, ≥5 drinks on the same occasion for males] on 5 or more days per month), “moderate” alcohol use (≥2 drinks per day for females, ≥3 drinks per day for males, or binge drinking ≥2 days per month, or a history of daily binge drinking), or “mild” alcohol use (not meeting the above criteria) (Rattan et al., 2022).

### 2.5. Statistical analyses

For all individuals in the present study, descriptive analyses were performed on the characteristics of each participant. Statistical analysis was conducted using R open-source software version 4.2.2 to analyze the data for this study. For continuous variables, the mean and standard deviation (SD) were reported, while for categorical variables, the percentages were reported. For continuous variables, the baseline characteristics were analyzed using a linear regression model, and for categorical variables, they were analyzed using a chi-square test. In this study, we employed the PSM method with a 1:2 ratio to balance cases and controls. This ratio is widely used in propensity score matching to increase statistical power and minimize potential bias in observational studies (Niu et al., 2021; Tang et al., 2021; Galvain et al., 2022). Univariate logistic analysis and multivariable logistic analysis were used to determine whether DII is related to PD. In the models, DII was treated as a continuous variable as well as categorized into tertiles, with tertile 1 serving as a reference group before and after PSM. We also conducted subgroup analyses based on age, gender, race, education level, family income, smoking status, alcohol consumption, strokes, diabetes, and heart disease before and after PSM. RCS analyses based on multivariable logistic analysis in model 3 were used to assess the non-linear association between DII and PD before and after matching. We considered all survey sampling weights when analyzing the data. It was considered statistically significant if *p* < 0.05.

## 3. Results

### 3.1. Demographic characteristics of participants

**Figure 2:**
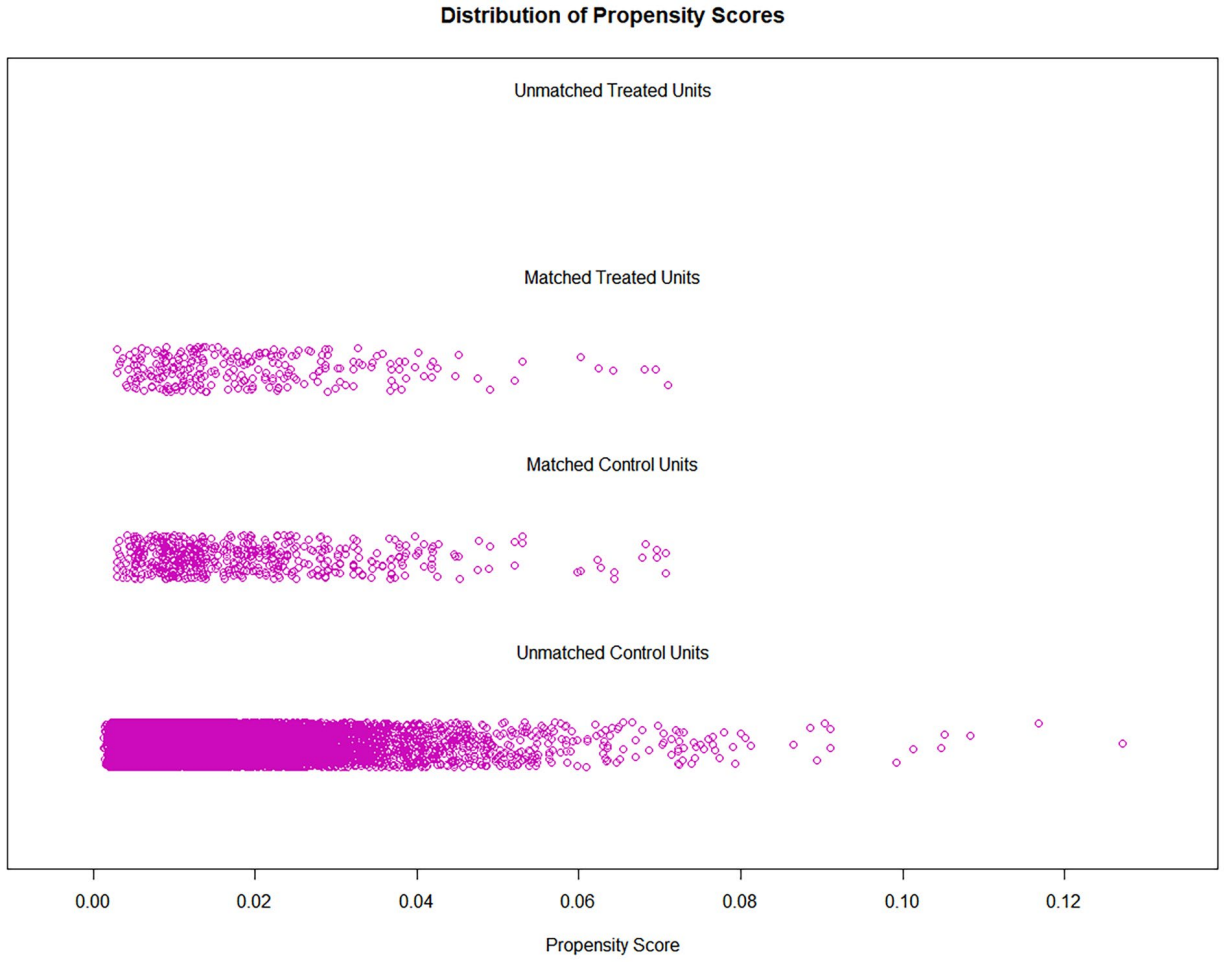
“Jitter” diagram of PSM legend (not application).

Between 2003 and 2018, a grand total of 80,241 participants took part in NHANES (Figure 1). The study excluded participants under 40 years of age based on the epidemiological characteristics of PD. For the calculation of DII or other variables, we excluded participants who did not provide essential dietary information; statistical analysis was conducted on 21,994 participants. The study included 263 patients with PD and 21,731 participants who did not have the disease. In Table 1, we show the general characteristics of the study population based on whether they had PD. It was found that people with PD were more likely to be older, women, non-Hispanic white, active drinkers, with lower family incomes, a higher DII, high waist circumference, and with a history of stroke or diabetes compared to non-PD individuals. We found that the DII mean (SE) and range in our study population were 0.029 (0.152) and 1.378–1.979, respectively. To further support the relationship between DII and PD risk, a comparison control group was established using nearest neighbor propensity score matching (PSM) (1:2). After PSM, there were no significant differences in the majority of baseline characteristics between the two groups, which matched 526 participants in the control group and 263 participants in the PD group (Table 2), however, DII was still significant differences between two groups. Figure 2 shows the data distribution before and after matching.

**Table 1:** Characteristics of the study population from NHANES 2003–2018 before matching.

Characteristic	Unmatching			
	Total (*n* = 21,994)	Non-PD (*n* = 21,731)	PD (*n* = 263)	*p*-value
Age	57.311 (0.164)	57.274 (0.163)	60.455 (1.089)	0.004**
Gender				0.003**
Male	10,891 (49.518)	10,774 (47.853)	117 (35.749)	
Female	11,103 (50.482)	10,957 (52.147)	146 (64.251)	
Race				0.006**
Non-Hispanic White	10,515 (47.808)	10,342 (74.617)	173 (83.171)	
Non-Hispanic Black	4,618 (20.997)	4,582 (9.834)	36 (7.583)	
Mexican American	3,265 (14.845)	3,239 (5.828)	26 (3.654)	
Other	3,596 (16.35)	3,568 (9.720)	28 (5.593)	
Education				0.401
Below high school	5,784 (26.298)	5,707 (15.635)	77 (18.922)	
High School	5,121 (23.284)	5,061 (24.134)	60 (25.725)	
Over high school	11,089 (50.418)	10,963 (60.231)	126 (55.354)	
Annual family income (USD)				< 0.001***
<20,000	5,394 (24.525)	5,299 (15.223)	95 (26.246)	
> = 20,000	16,600 (75.475)	16,432 (84.777)	168 (73.754)	
BMI (kg/m2)	29.350 (0.075)	29.340 (0.076)	30.214 (0.570)	0.131
Waist (cm)	101.435 (0.188)	101.398 (0.190)	104.600 (1.275)	0.015*
DII	1.385 (0.029)	1.378 (0.029)	1.979 (0.152)	< 0.001***
Smoke				0.062
Never	11,093 (50.436)	10,963 (51.146)	130 (50.907)	
Former	6,793 (30.886)	6,720 (30.874)	73 (24.953)	
Now	4,108 (18.678)	4,048 (17.980)	60 (24.141)	
Alcohol				0.029*
Never	3,158 (14.358)	3,121 (10.707)	37 (10.912)	
Former	4,722 (21.469)	4,639 (17.434)	83 (26.951)	
Mild	8,073 (36.705)	7,981 (41.246)	92 (37.272)	
Moderate	2,975 (13.526)	2,951 (16.108)	24 (13.218)	
Heavy	3,066 (13.94)	3,039 (14.505)	27 (11.648)	
Stroke				< 0.0001***
No	20,829 (94.703)	20,599 (96.095)	230 (88.680)	
Yes	1,165 (5.297)	1,132 (3.905)	33 (11.320)	
Diabetes				0.017*
No	16,758 (76.194)	16,581 (81.937)	177 (74.923)	
Yes	5,236 (23.806)	5,150 (18.063)	86 (25.077)	
Heart attack				0.093
No	20,598 (93.653)	20,364 (94.955)	234 (92.264)	
Yes	1,396 (6.347)	1,367 (5.045)	29 (7.736)	

Data are presented as N% (χ^2^-test) or Mean (SD) (independent *t*-test). *p < 0.05, **p < 0.01, ***p < 0.001. Bold values indicates *p* < 0.05 signifying statistical significance.

**Table 2:** Characteristics of the study population from NHANES 2003–2018 after matching.

Characteristic	Matching			
	Total (*n* = 789)	Non-PD (*n* = 526)	PD (*n* = 263)	*P*-value
Age	61.004 (0.519)	61.238 (0.528)	60.455 (1.089)	0.5
Gender				0.936
Male	353 (44.74)	236 (42.285)	117 (35.749)	
Female	436 (55.26)	290 (57.715)	146 (64.251)	
Race				0.716
Non-Hispanic White	516 (65.399)	343 (86.561)	173 (83.171)	
Non-Hispanic Black	119 (15.082)	83 (5.958)	36 (7.583)	
Mexican American	78 (9.886)	52 (2.615)	26 (3.654)	
Other	76 (9.632)	48 (4.866)	28 (5.593)	
Education				0.844
Below high school	219 (27.757)	142 (15.285)	77 (18.922)	
High School	185 (23.447)	125 (26.341)	60 (25.725)	
Over high school	385 (48.796)	259 (58.374)	126 (55.354)	
Annual family income (USD)				0.924
<20,000	287 (36.375)	192 (21.253)	95 (26.246)	
> = 20,000	502 (63.625)	334 (78.747)	168 (73.754)	
BMI (kg/m2)	30.781 (0.380)	31.023 (0.469)	30.214 (0.570)	0.264
Waist (cm)	106.112 (0.939)	106.757 (1.183)	104.600 (1.275)	0.204
DII	1.656 (0.099)	1.519 (0.120)	1.979 (0.152)	0.015*
Smoke				0.598
Never	412 (52.218)	282 (60.306)	130 (50.907)	
Former	175 (22.18)	115 (18.112)	60 (24.141)	
Now	202 (25.602)	129 (21.581)	73 (24.953)	
Alcohol				0.82
Never	124 (15.716)	87 (13.541)	37 (10.912)	
Former	246 (31.179)	163 (24.263)	83 (26.951)	
Mild	279 (35.361)	187 (46.193)	92 (37.272)	
Moderate	73 (9.252)	49 (9.628)	24 (13.218)	
Heavy	67 (8.492)	40 (6.376)	27 (11.648)	
Stroke				0.895
No	688 (87.199)	458 (92.111)	230 (88.680)	
Yes	101 (12.801)	68 (7.889)	33 (11.320)	
Diabetes				1
No	531 (67.3)	354 (71.563)	177 (74.923)	
Yes	258 (32.7)	172 (28.437)	86 (25.077)	
Heart attack				0.942
No	703 (89.1)	469 (92.341)	234 (92.264)	
Yes	86 (10.9)	57 (7.659)	29 (7.736)	

Data are presented as N% (*χ*^2^-test) or Mean (SD) (independent *t*-test). *p < 0.05, **p < 0.01, ***p < 0.001. Bold values indicates *p* < 0.05 signifying statistical significance.

### 3.2. Association between DII and PD

#### 3.2.1. Univariate logistics regression analysis of the association between DII and PD

Univariate logistics analysis was used for observation of the associations between age, gender, race, education level, BMI, annual family income, waist circumference, smoking, alcohol, stroke, diabetes mellitus, and heart attack in the US population. According to our survey, age positively correlated with PD occurrence, and the odds ratio (OR) and 95% confidence interval (CI) was 1.02 (1.01, 1.04). There was a higher likelihood of women developing PD compared to men [OR: 1.65; 95%CI: (1.19, 2.29)]. Families with low income (less than 2000 dollars per year) had a lower incidence of PD, with a relative OR and 95% confidence interval of 0.50 (0.35, 0.73). There was a higher risk of PD in people who had had a stroke and diabetics as compared to people who had not had a stroke and non-diabetics [OR:1.52; 95%CI: (1.08–2.14) and OR:1.58; 95%CI: (0.93, 2.70), respectively]. Meanwhile, we found that DII was positively associated with the occurrence of PD [OR: 1.21 (1.09, 1.34)] (*p* < 0.001). As shown in Table 3, the OR for the correlation of DII with PD was 2.20 (1.50, 3.23) (*p* < 0.0001) in group T3 compared with group T1, and T2 groups were not statistically different. And after matching, the association of DII with PD was also positive [OR: 1.156 (1.025, 1.303)] (*p* < 0.05) as a continuous variable and [OR:2.012 (1.281, 3.159)] (*p* < 0.05) in group T3 compared with group T1 as categorized into tertiles.

**Table 3:** Univariate logistics regression analysis of the association between DII and PD.

Characteristic	Unmatching		Matching	
	OR (95%CI)	*P*-value	OR (95%CI)	*P*-value
Age	1.02 (1.01, 1.04)	0.003**	0.994 (0.977, 1.011)	0.503
Gender				
Male	ref	ref	ref	ref
Female	1.65 (1.19, 2.29)	0.003**	1.317 (0.884, 1.961)	0.173
Race				
Non-Hispanic White	ref	ref	ref	ref
Non-Hispanic Black	0.69 (0.46, 1.04)	0.08	1.325 (0.805, 2.180)	0.266
Mexican American	0.56 (0.35, 0.90)	0.02*	1.454 (0.811, 2.608)	0.207
Other	0.52 (0.29, 0.91)	0.02	1.196 (0.545, 2.627)	0.653
Education				
Below high school	ref	ref	ref	ref
High School	0.88 (0.54, 1.43)	0.60	0.789 (0.436, 1.428)	0.430
Over high school	0.76 (0.51, 1.14)	0.18	0.766 (0.490, 1.197)	0.239
Annual family income (USD)				
<20,000	ref	ref	ref	ref
> = 20,000	0.50 (0.35, 0.73)	<0.001***	0.758 (0.513, 1.120)	0.163
BMI (kg/m2)	1.02 (1.00, 1.04)	0.10	0.982 (0.951, 1.014)	0.261
Waist (cm)	1.01 (1.00, 1.02)	0.01**	0.992 (0.980, 1.004)	0.194
DII	1.21 (1.09, 1.34)	<0.001***	1.156 (1.025, 1.303)	0.018*
DII T1	ref	ref	ref	ref
DII T2	1.37 (0.90, 2.09)	0.14	1.251 (0.734, 2.133)	0.407
DII T3	2.20 (1.50, 3.23)	<0.0001***	2.012 (1.281, 3.159)	0.003**
Smoke				
Never	ref	ref	ref	ref
Former	0.81 (0.57, 1.15)	0.24	1.370 (0.855, 2.195)	0.189
Now	1.35 (0.92, 1.98)	0.13	1.579 (1.030, 2.420)	0.036
Alcohol				
Never	ref	ref	ref	ref
Former	1.52 (0.94, 2.45)	0.09	1.378 (0.755, 2.517)	0.293
Mild	0.89 (0.55, 1.42)	0.61	1.001 (0.565, 1.775)	0.996
Moderate	0.81 (0.40, 1.60)	0.53	1.704 (0.744, 3.899)	0.205
Heavy	0.79 (0.40, 1.54)	0.48	2.267 (0.885, 5.808)	0.087
Stroke	ref	ref		
No	0.50 (0.35, 0.73)	<0.001***	ref	ref
Yes	1.01 (1.00, 1.02)	0.01**	1.490 (0.829, 2.679)	0.180
Diabetes				
No	ref	ref	ref	ref
Yes	1.52 (1.08, 2.14)	0.02*	0.842 (0.530, 1.338)	0.464
Heart attack				
No	ref	ref	ref	ref
Yes	1.58 (0.92, 2.70)	0.10	1.011 (0.535, 1.911)	0.973

*p < 0.05, **p < 0.01, ***p < 0.001. Bold values indicates *p* < 0.05 signifying statistical significance.

#### 3.2.2. Multivariable logistics regression analysis of the association between DII and PD

As shown in Table 4, three logistic regression models were constructed to analyze the relationship between DII and PD in American adults. Model 1 was a crude model without covariates adjusted. Model 2 was adjusted by age, gender, and race. Model 3 was adjusted by age, gender, race, education level, BMI, annual family income, waist circumference, smoking status, alcohol, stroke, diabetes, and heart attack. We found that DII was positively associated with the risk of PD in all models with OR and 95%CI of 1.209 (1.090, 1.341), 1.187 (1.064, 1.324), and 1.129 (1.013,1.259) (*p* < 0.05), respectively. All models were significantly different at T3 with OR and 95%CI of 2.200 (1.499, 3.229), 2.027 (1.349, 3.045), and 1.666 (1.099, 2.525) (*p* < 0.005) as categorized into tertiles. Using our survey as a comparison, we found that higher DII levels may have been an independent risk factor for PD. A significant association between high DII levels and increased PD was still observed after matching in three models, even though the association was somewhat weakened.

**Table 4:** Multivariable logistics regression analysis of the association between DII and PD.

Model	Characteristic	Unmatching		Matching	
		OR (95%CI)	*P*-value	OR (95%CI)	*P*-value
Model 1	Total	1.209 (1.090, 1.341)	<0.0001***	1.156 (1.025, 1.303)	0.018*
	T1	ref	ref	ref	ref
	T2	1.370 (0.899, 2.089)	0.142	1.539 (0.951, 2.491)	0.407
	T3	2.200 (1.499, 3.229)	<0.0001***	1.856 (1.194, 2.884)	0.003**
Model 2	Total	1.187 (1.064, 1.324)	0.002**	1.139 (1.005, 1.291)	0.042*
	T1	ref	ref	ref	ref
	T2	1.316 (0.859, 2.014)	0.205	1.222 (0.706, 2.115)	0.469
	T3	2.027 (1.349, 3.045)	<0.001***	1.906 (1.180, 3.078)	0.009**
Model 3	Total	1.129 (1.013, 1.259)	0.029*	1.168 (1.038, 1.315)	0.01*
	T1	ref	ref	ref	ref
	T2	1.220 (0.800, 1.861)	0.0352	1.339 (0.775, 2.315)	0.292
	T3	1.666 (1.099, 2.525)	0.017*	2.071 (1.277, 3.358)	0.004**

Model 1: No adjustments made for confounding factors. Model 2: Adjustments made for age, sex, and race. Model 3: Adjustments made for age, gender, race, education level, BMI, annual family income, waist circumference, smoking status, alcohol, stroke, diabetes, and heart attack. *p < 0.05, **p < 0.01, ***p < 0.001. Bold values indicates *p* < 0.05 signifying statistical significance.

#### 3.2.3. Subgroup analysis before and after matching

**Figure 3:**
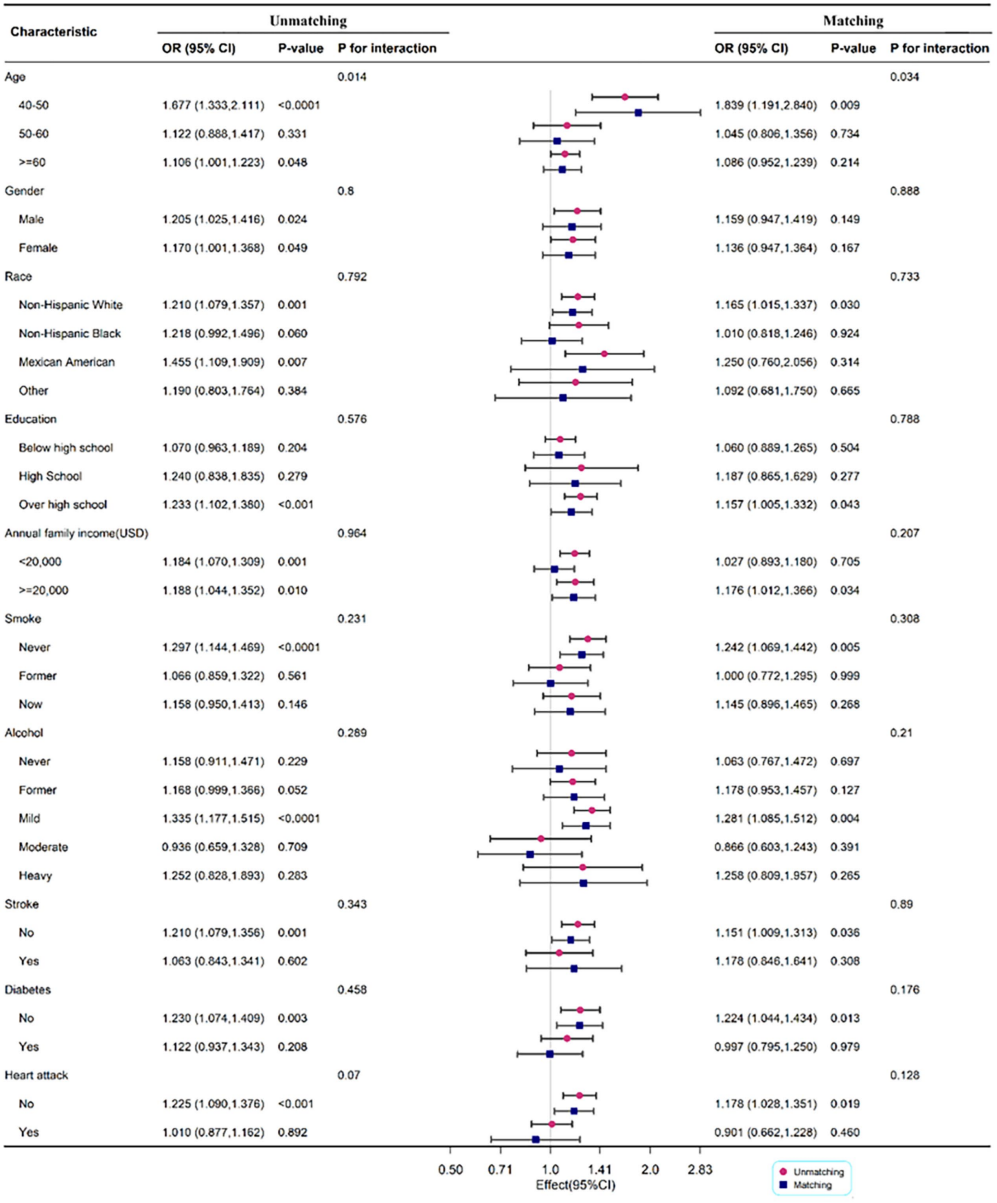
Subgroup analysis for the association between DII and PD legend: subgroup analysis for the association between DII and PD. Weighted univariate logistic regression was used for subgroup analysis.

To determine the relationship between DII and PD by age, gender, race, annual family income, smoking status, alcohol, stroke, diabetes, and heart attack, subgroup analysis was employed. Age may influence the positive association between DII and PD (*P* for interaction <0.05). The correlation between DII and PD was more significant in men (OR: 1.205; 95%CI: 1.025–1.416) before matching, but there was no significant difference between the two groups after matching. When race was used in the subgroup analysis, the correlation was more substantial in non-Hispanic white with OR 1.210 (1.079, 1.357) and 1.165 (1.015, 1.337) before and after matching, respectively (Figure 3).

#### 3.2.4. Nonlinear associations between DII and the risk of PD

**Figure 4:**
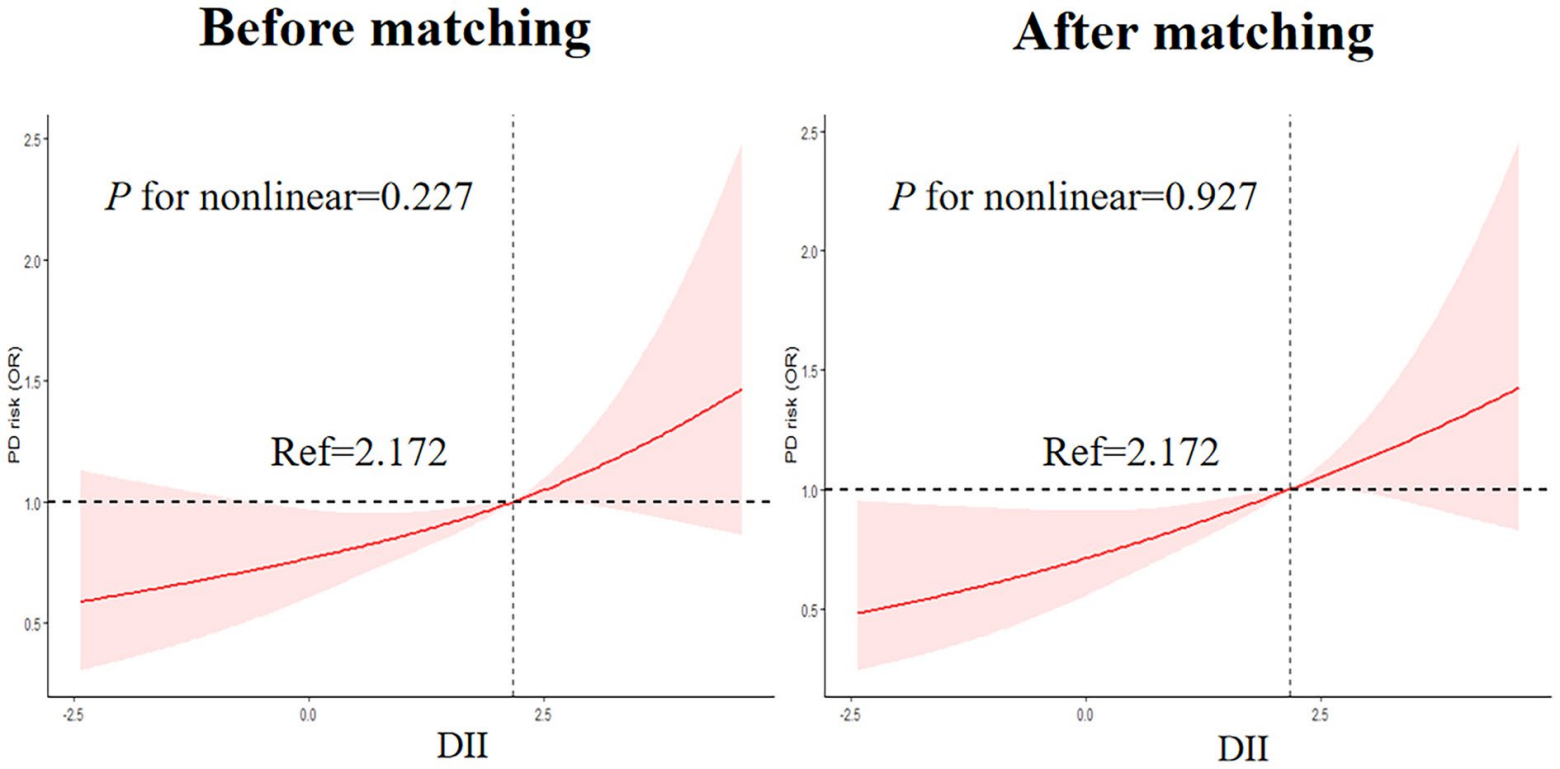
Nonlinear Associations between DII and the risk of PD legend: RCS before and after matching based on Model 3.

As shown in Figure 4, RCS was drawn to visually describe the relationship between DII and PD before and after matching based on Model 3 (Figure 4). However, the results of the RCS analysis revealed no nonlinear associations between DII and PD (*p* for nonlinear =0.227 and 0.927, before and after matching, respectively).

## 4. Discussion

Based on NHANES data from 2003 to 2018, we investigated the relationship between PD and DII among US adults above 40. We found that there was a significant difference in DII levels between participants with PD and those without PD diagnoses. We also found that the DII was positively associated with the prevalence of PD. Univariate and multivariate logistic regression analysis showed associations between DII and PD. Additionally, individuals in males or non-Hispanic whites were more likely to experience the progression of PD with an increase in DII. Besides, the RCS analysis showed no nonlinear associations between DII and PD.

PD is a chronic progressive neurological disorder characterized by the degeneration of dopaminergic neurons (Tolosa et al., 2021). As is increasingly recognized, inflammation plays a crucial role in a wide range of pathological conditions and chronic diseases (Liu et al., 2021b; Wu et al., 2022). Numerous studies have investigated the relationship between PD and inflammation. A meta-analysis showed that the TGF-β1, IL-6, and IL-1β levels were significantly increased in patients with PD when compared with controls in cerebrospinal fluid (Chen et al., 2018). There is a positive correlation between high-sensitivity C-reactive protein and PD risk (Baran et al., 2019; Jin et al., 2020). To conclude, inflammation plays a significant role in PD occurrence and development.

Oxidative stress is another important mechanism in the development of PD (Subramaniam and Chesselet, 2013). It arises from an imbalance between the generation of reactive oxygen species (ROS) and the body’s antioxidant defense system. ROS can inflict damage upon vital cellular components, such as proteins, lipids, and DNA, ultimately leading to neurodegeneration (Subramaniam and Chesselet, 2013). Unhealthy dietary factors, including excessive consumption of fats and inadequate intake of antioxidants from fruits and vegetables, can contribute to heightened oxidative stress (Miranda-Díaz et al., 2020). This oxidative stress can expedite the demise of dopaminergic neurons, a characteristic hallmark of PD (Subramaniam and Chesselet, 2013). Notably, studies have demonstrated a correlation between an elevated DII index and markers of oxidative stress (Zhang et al., 2022). This finding suggests that the dietary inflammation index score also reflects the body’s oxidative stress, and consuming inflammatory foods can inflict nerve damage through this mechanism.

Meanwhile, it has been found that diet has a significant role in modulating the progression and development of PD. The effects of different dietary interventions on PD symptoms have been demonstrated, with nutrition and diet representing modifiable risk factors for reducing disease risk (Maswood et al., 2004; Jabre and Bejjani, 2006). There is evidence from a prospective study of 805 subjects followed for 20–22 years that some flavonoids may reduce the risk of PD (Gao et al., 2012). According to a population-based cohort study of 1731 elder adults from Greece, those who adhered more to the Mediterranean diet had a lower risk of prodromal PD (Maraki et al., 2019). There is evidence that dairy foods might be positively associated with an increased risk of PD, according to a meta-analysis of prospective cohort studies including 1,083 PD cases among 304,193 participants (Jiang et al., 2014). A prospective population-based cohort study among 5,289 subjects reported that intakes of total fat, monounsaturated fatty acids, and polyunsaturated fatty acids were significantly associated with a lower risk of PD (de Lau et al., 2005). Researchers found that fresh vegetables, fresh fruit, nuts and seeds, non-fried fish, olive oil, wine, coconut oil, fresh herbs, spices, coenzyme Q10, and fish oil reduced the progression of PD, while canned fruits and vegetables, diet sodas, fried food, beef, ice cream, yogurt, cheese, and iron are associated with more rapid progression of PD in a prospective observational study of 1,053 individuals (Mischley et al., 2017). It has been shown that a higher intake of antioxidant-rich foods may reduce the risk of Parkinson’s disease in a systematic review (Talebi et al., 2022).

To our knowledge, there have been no studies investigating the association between the inflammatory potential of diet, assessed using an easy-to-use tool like the DII, and PD.

In recent years, DII has attracted much scientific interest as a tool for measuring the inflammatory potential of an individual’s diet in different neurodegenerative disorders (Balomenos et al., 2022). Diets with a negative DII score have an anti-inflammatory effect that protects against PD, while diets with a positive score have a pro-inflammatory effect. The current study showed significant increases in DII levels in patients with PD based on 28,041 adults from the NHANES. A univariate and multivariable logistic regression analysis was performed to investigate the association between DII and PD and RCS was also used to describe their collection. There was an increased risk of PD associated with high DII either as continuous variables or as categorical variables grouped by tertile. As a result of our study, we suggest that people take in an anti-inflammatory diet, like the Mediterranean diet, rather than a pro-inflammatory diet, like the Standard American Diet, to prevent and treat PD.

Our study has several advantages and implications. It is worth noting that the analysis incorporates the use of sampling weights assigned to each participant. These weights play a crucial role in enabling statistical inferences and generalizing our findings to a larger population beyond the sample size of PD cases. By accounting for these weights, our study ensures reliable conclusions and precise statistical power. In addition, our study used PSM to eliminate confounding factors, improving the credibility of the results. Third, a stratified subgroup analysis was conducted to further investigate the relationship between DII and PD across different population groups, which suggests that we need to implement more precise prevention strategies for PD.

This study also has several limitations that need to be clarified. In the first place, it should be noted that in our study, PD is defined based on self-reported use of prescription drugs, rather than a clinical diagnosis by a healthcare professional. In our study, we acknowledge the possibility of some individuals being unaware of their PD diagnosis or having milder PD symptoms that do not require medication. Additionally, we recognize that there may be cases where patients with other neurological disorders exhibiting tremors are prescribed antiparkinsonian therapy without having a definitive diagnosis of PD. As a result, there is a potential for misclassification, which may introduce bias into our results. Future research should aim to incorporate more rigorous diagnostic criteria, such as clinical evaluations conducted by movement disorder specialists or the utilization of standardized diagnostic tests. By implementing these measures, it will be possible to accurately identify cases of PD and minimize the potential for misclassification It is also important to enhance the validation of alternative methods for identifying PD cases beyond self-reporting by incorporating medical records or employing more comprehensive diagnostic tools. Through this, we can ensure a more robust assessment of the association between dietary patterns and PD. Additionally, causal associations could not be determined due to the cross-sectional nature of the research. For a more accurate understanding of the relationship between DII and PD, further prospective studies are needed. There was a challenge in excluding bias due to the absence of other confounders in this study.

## 5. Conclusion

Our study found DII was associated with PD, and we explored subgroup differences. In addition to having major implications for clinical practice, our findings have important public health implications as well. It is possible to prevent and reduce PD by restricting a pro-inflammatory diet or encouraging an anti-inflammatory diet because the diet is a factor that can be changed. It is necessary to conduct further basic studies to determine whether DII affects PD.

## Data availability statement

Publicly available datasets were analyzed in this study. This data can be found at: https://www.cdc.gov/nchs/nhanes/index.htm.

## Ethics statement

The studies involving human participants were reviewed and approved by the NCHS Research Ethics Review Board. The patients/participants provided their written informed consent to participate in this study.

## Author contributions

ZZ designed the study. YC and ZZ wrote the manuscript. YC collected and analyzed the data. LW interpreted the data. XL reviewed, edited, and approved the manuscript. All authors read and approved the final manuscript.

## Funding

This work was supported by the Science and Technology Planning Project of Shenzhen Municipality (KCXFZ20201221173605013).

## Conflict of interest

The authors declare that the research was conducted in the absence of any commercial or financial relationships that could be construed as a potential conflict of interest.

## Publisher’s note

All claims expressed in this article are solely those of the authors and do not necessarily represent those of their affiliated organizations, or those of the publisher, the editors and the reviewers. Any product that may be evaluated in this article, or claim that may be made by its manufacturer, is not guaranteed or endorsed by the publisher.
